# Proof of Kernel Work: a democratic low-energy consensus for distributed access-control protocols

**DOI:** 10.1098/rsos.180422

**Published:** 2018-08-08

**Authors:** Leif-Nissen Lundbæk, Daniel Janes Beutel, Michael Huth, Stephen Jackson, Laurence Kirk, Robert Steiner

**Affiliations:** 1 Imperial College London, London SW7 2AZ, UK; 2 Oxford University, Oxford OX1 2JD, UK; 3 XAIN Research, Schmiedestraße 2, 15745 Wildau, Germany

**Keywords:** Cryptographic Sortition, access control, blockchain, cybersecurity

## Abstract

We adjust the Proof of Work (PoW) consensus mechanism used in Bitcoin and Ethereum so that we can build on its strength while also addressing, in part, some of its perceived weaknesses. Notably, our work is motivated by the high energy consumption for mining PoW, and we want to restrict the use of PoW to a configurable, expected size of nodes, as a function of the local blockchain state. The approach we develop for this rests on three pillars: (i) *Proof of Kernel Work* (PoKW), a means of dynamically reducing the set of nodes that can participate in the solving of PoW puzzles such that an adversary cannot increase his attack surface because of such a reduction; (ii) *Practical Adaptation of Existing Technology*, a realization of this PoW reduction through an adaptation of existing blockchain and enterprise technology stacks; and (iii) *Machine Learning for Adaptive System Resiliency*, the use of techniques from artificial intelligence to make our approach adaptive to system, network and attack dynamics. We develop here, in detail, the first pillar and illustrate the second pillar through a real use case, a pilot project done with Porsche on controlling permissions to vehicle and data log accesses. We also discuss pertinent attack vectors for PoKW consensus and their mitigation. Moreover, we sketch how our approach may lead to more democratic PoKW-based blockchain systems for public networks that may inherit the resilience of blockchains based on PoW.

## 1. Introduction

There is little doubt that blockchains, such as Bitcoin created in 2008, have had a significant impact on the global start-up scene, FinTech, asset managers and traders, regulators and policy-makers, as well as on academics working in distributed systems, cybersecurity and economics.

But the intense growth of blockchain systems and cryptocurrencies has also led to a perceived hype and deplorable misconceptions of the underlying technologies and their potential. For example, commercial entities have been deploying services based on distributed ledger technology (DLT) since the 1990s—based on the concept of a hash chain. And many perceived use cases of blockchain may already be adequately covered by such existing DLT technology, with little or no need for more recent blockchain innovations. In fact, one may say that—for a particular use case—a blockchain is the right choice over a hash-chain-type DLT solution whenever there is

C1 a concern about the ability of insiders to maliciously or unintentionally facilitate an attack of the decentralized data management system realized by this blockchain or
C2 it is desired or mandatory that the re-writing of part of the data history would require considerable effort that is quantifiable in terms of cost, time or other measures of interest.

System requirements and use cases that do not have such concerns may often be well catered for with DLT-based systems that do not use hardening mechanisms such as cryptographic puzzles and their solutions in order to address concerns C1 and C2 above. By contrast, our work reported in this paper aims at developing blockchain technology that can harden system resiliency by addressing these concerns. For example, the networks that one would intend to deploy in intelligent transportation systems may be open and thus exposed to attacks on their confidentiality, integrity and availability; and even their closed parts may be subject to insider attacks that could bring down important functionality or compromise information security.

One major attack surface of a blockchain is its chosen consensus mechanism. There are many proposals for such mechanisms in the literature, including those that rest on classical Byzantine fault-tolerant consensus protocols (e.g. [1]). Some others are rooted in the developments of blockchain technology; see [2] or §2.5.3 in [3] for an overview of the most prominent such BCT-based proposals. Yet, these approaches either have severe scalability issues or fail to adequately address concerns C1 and C2. Very recent conceptual developments, such as Hedera's Hashgraph [4], still need to be validated by their implementation and operation. In that light, *cryptographic puzzles* (e.g. ch. 8 in [5]) appear to be an attractive alternative. The most promising such cryptographic puzzle is perhaps Proof of Work (PoW), as used in Bitcoin and Ethereum, whereas alternative puzzle definitions seem to be ill-suited for needs of real-world blockchain systems.

In simplified terms, the idea behind PoW is that the puzzle contains specified information *i* about the current blockchain, notably its last block, and that one needs to add some random source *r* to that information in order for a hash *h*(*i*||*r*) of the concatenation of *i* and *m* to have a certain number of leading zeros.

The cryptographic properties of hash function *h* are what makes this a hard puzzle to solve: there is no way of predicting, given *i* and *h*, a value of *r* that is more likely to solve that puzzle than any other value. The hash function *h* should also be chosen so that there is no genuine advantage in trying to solve this puzzle with a random source *r*_*k*_ if one has already failed to solve it for random sources *r*_0_ up to *r*_*k*−1_ for the same information *i*. That way, anyone who joins a race to solve a puzzle has the same chance of winning that race—regardless of how long others have already tried to win it.

PoW thus allows any node in a network to participate in such a concurrent mining race, where it is impossible to predict who will first find a solution to the current puzzle, the information encoded in *i*. It is this unpredictability that makes PoW so secure. The winner of this race, announced on the peer-to-peer network, is then the elected leader whose solution determines the next block on the chain.

Although this election process may have local divergence on who the leader is, a PoW blockchain corrects such divergence since each node keeps a ‘blocktree’ in which exactly one path represents the blockchain. Such divergence is thus recorded in that tree as a different path. The network eventually self-stabilizes, since each node interprets the deterministically chosen path of its tree that represents the most overall work as the ‘true’ blockchain.

A major concern about this approach, however, is that solving a PoW puzzle is power hungry, many hashes have to be computed before a solution may be found. Moreover, incentive structures for puzzle solving may result in more and more nodes joining such mining races, leading to an even greater consumption of energy on the network in order to sustain a PoW consensus mechanism. These problems are made worse for blockchains that mint coins which can be traded for fiat currencies, and where the level of difficulty may have to increase in order to reflect increasing computational power of puzzle solvers.

A good example thereof is Bitcoin, where application-specific integrated circuits for PoW were developed in record time and are widely available commercially. This meant that the PoW mining race was no longer democratic as only such high-end hardware made it viable to compete in the race, and only if individual devices/agents would pool their resources in such races. This, combined with the speculation around Bitcoin trading platforms, has meant that the Bitcoin network consumes, at the time of writing, as much energy as the entire nation of Sweden. PoW mining races in Bitcoin, therefore, seem to be undemocratic and contribute to global warming.

These issues motivate us to ask whether one could retain the strong aspects of PoW (notably its resilient and random manner of choosing the leader for the next block), while also weakening or eliminating the disadvantages of PoW discussed above.

Our contributions in this paper are a result of trying to answer this question and are as follows:

— we propose a variant of PoW, Proof of Kernel Work (PoKW), based on Cryptographic Sortition [6,7] that dynamically reduces the mining race to a small kernel of randomly selected nodes,
— we show how our PoKW reduces the energy demands of puzzle solving whilst also not increasing abilities of an attacker in an enterprise network,
— we identify pertinent attack vectors for blockchains based on PoKW and mitigation measures for such attacks,
— we sketch possible ways of realizing PoKW-based public networks, in order to create more democratic yet resilient infrastructures for blockchain-facilitated services, and
— we adjust an Ethereum technology stack to accommodate this novel consensus mechanism and report insights from a real-world use case of that system.

The PoKW expresses the reduction of mining races to a randomly chosen set of network nodes (the kernel). It should be stressed that PoKW does not rely on use of a particular blockchain technology stack, making PoKW applicable within a broad range of blockchain solutions.

*Outline of paper*: In §2, we present our modified PoW, PoKW. In §3, we discuss how access control may be managed for PoKW-based blockchains. The potential of PoKW to support more democratic, public blockchain networks is the subject of §4. In §5, the attack surface of PoKW is explored and mitigations discussed. A real use case of a PoKW-based blockchain is featured in §6 and the paper concludes in §7.

## 2. Proof of Kernel Work

PoW, as currently used in Bitcoin and Ethereum, has been very successful as a consensus mechanism for cryptocurrencies and blockchain systems. However, as discussed above, PoW consumes a lot of energy and leads to centralization of mining in standard incentivization structures. Therefore, it seems desirable to retain the advantages of PoW while also containing its energy consumption and mitigating, if not eliminating, centralization of mining. This paper reports our proposal for adapting PoW to achieve such desirable system properties.

Let us first point out related work and how it informs our approach. The work in [8] already developed means of minimizing energy consumption of PoW in the ‘governed blockchain’ setting [9], at a guaranteed level of security. That setting is suitable for enterprise blockchains, since miners are procured resources owned or controlled by an organization or consortium. It also comes with a mathematical model that can compute initial system configurations, so that optimal trade-offs between security, cost and availability are found.

The approach we develop in this paper can make use of such optimization, as sketched below. But we here mean to focus on how we control participation in the PoW mining race so that this control mechanism cannot be corrupted by a powerful adversary. We also mean to discuss how this approach can, in principle, by adopted on public networks in which any network node may join activities in order to realize a more democratic network structure. Throughout the paper, we emphasize conceptual structures (e.g. which new components to add to the structure of blocks) over routine implementation details (such as specific hash trees for transaction sets).

Conceptually, a blockchain *B*^0^, *B*^1^, …, *B*^*r*−1^ consists of a linearly ordered list of blocks, where block *B*^*i*^ has block number *i* and block height *i* − 1. The latter refers to the number of blocks that precede *B*^*i*^ in that chain. Block *B*^0^ is the *Genesis Block*. Each block *B*^*r*^ with *r* > 0 is determined by a *mining race* that also makes *B*^*r*^ depend on *B*^*r*−1^.

### 2.1. Intuition behind Proof of Kernel Work

**Figure 1.**
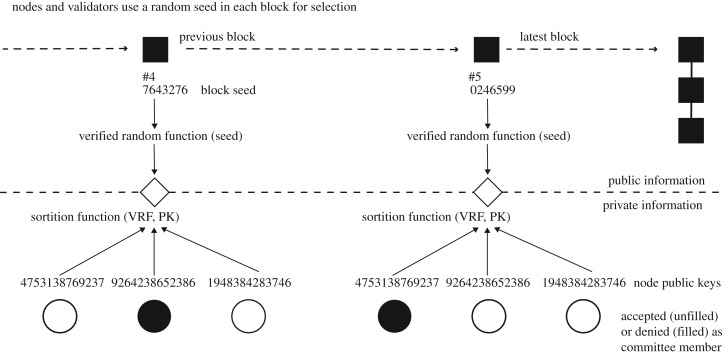
An illustration of the Cryptographic Sortition process used in PoKW.

In our approach, the process of electing a leader—who can propose the next block on the chain—relies on PoW. However, we restrict the PoW mining race for the next block by two control mechanisms:

WL A dynamic White List *L* which is authenticated on the blockchain and maintains those public keys that are, in principle, eligible to participate in a PoW mining race.
CS An adaptive node selection mechanism, illustrated in figure 1, based on *Cryptographic Sortition* as introduced in Algorand [6,7].

Cryptographic Sortition determines the superset of nodes that may be eligible to participate in specific tasks of blockchain construction and management. Such tasks may include mining, machine learning, and management of the White List *L*. Below, we will make machine learning a task that is (periodically) subsumed by the task of mining. Our approach lends itself to other realizations of task divisions and dependencies, though. The task eligibility based on Cryptographic Sortition is necessary but not sufficient for engaging in a task: White List *L* or other security state may override such eligibility as discussed below.

The utility of the White List control mechanism is that it can support adaptive access control informed by anomaly detection, an aspect we will not develop here. The second control mechanism, based on Cryptographic Sortition:

— randomly selects, at each blockheight *r*≥1, from the set of all nodes on the White List *L* those nodes eligible to complete a task—such as mining a block *B*^*r*^
— this selection process is sufficiently random and only the selected nodes themselves will know that they are selected
— an adversary who can compromise nodes cannot exploit this selection mechanism to inform which nodes it aims to compromise
— the expected number of selected nodes, *n*_*w*_, is a parameter of the blockchain system.

This second mechanism, therefore, offers many advantages. For one, a system can control the expected number of nodes that participate in specific tasks such as mining or administering the White List *L*. This can save energy costs as fewer miners will lose a mining race. It can also modify the game theory of incentive structures for application domains that incorporate such incentives, for example, for cryptocurrencies. The pooling of mining resources, as seen in Bitcoin and other cryptocurrency blockchains, may no longer be effective or would require novel strategic pooling and reward-division behaviour. The apparent advantages resulting from this node selection mechanism apply equally to blockchain systems that are open (any node may join the system) or closed (only specified nodes are permitted to participate in the system).

Moreover, the blockchain has a system parameter *p*≥1. It ensures that all mining races of blockheights *r* that are multiples of *p* are also doing machine learning that will inform the modification of system parameters, whose changes would be embedded in the next block *B*^*r*^, or after a configuration-dependent number of subsequent blocks. Such learning would aim to detect anomalies and to ensure network stability.

Another important system parameter is the expected number of nodes that participate in mining races for the next *p* or so blocks. The actual set of nodes eligible to participate in a specific task at blockheight *r* will be a function of the White List *L* at blockheight *r* − 1, the local blockchain, and the superset generated by the second mechanism above.

### 2.2. Specification of Proof of Kernel Work

We will now specify PoKW, our modified PoW mechanism, in sufficient detail.

#### 2.2.1. Block structure.

Using the notation in [6,7], we let a block have *conceptual* structure

2.1 $$B^{r}=(r,{\mathrm{TSet}}^{r},Q^{r},H(B^{r-1}),{\mathrm{nonce}}^{r},k,p,n_{w},\ldots ),$$

where

— *r* is the blockheight of block *B*^*r*^,
— TSet^*r*^ is the payload, a set of transactions of the application domain,
— *Q*^*r*^ is the *seed* of block *B*^*r*^—a concept introduced in [6,7],
— *H*(*B*^*r*−1^) is the hash of the previous block *B*^*r*−1^,
— nonce^*r*^ is the value that demonstrates PoW for block *B*^*r*^,
— *k* is a security parameter similar to that used in Algorand [6,7],
— *p* specifies that machine learning is meant to happen every *p*th block,
— *n*_*w*_ is the expected number of nodes eligible to participate in a task for creating *B*^*r*^, and
— other components ‘…’ may contain additional configuration information.

**Figure 2.**
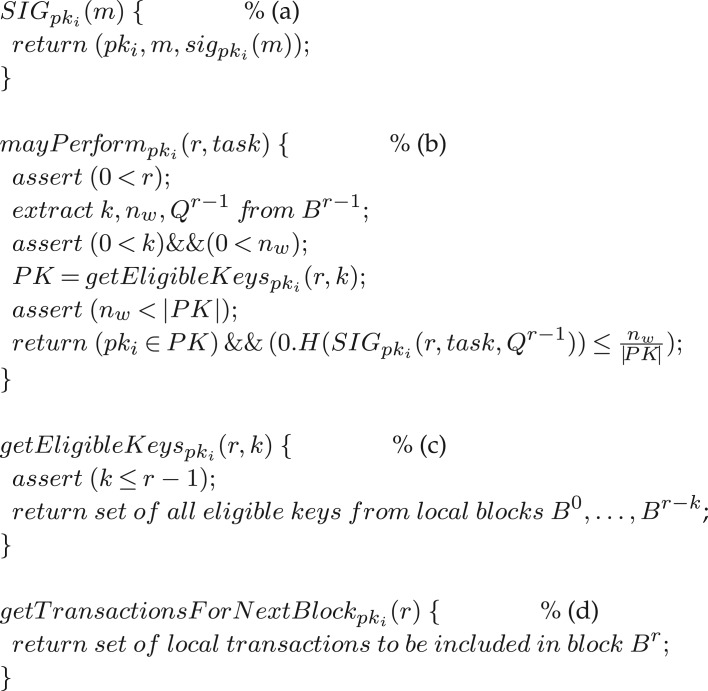
(a) Specification of digital signatures for node *pk*_*i*_, sig_pk_*i*__(*m*) refers to the digital signature of message *m* with private key for *pk*_*i*_. (b) Specification of checking whether node *pk*_*i*_ is a potential participant in the task *task*. (c) Function name for extraction of set of eligible public keys from local blockchain state. (d) Function name for computing local transactions that should get into the next block (should node *pk*_*i*_ become leader). The manner in which outputs for (c) and (d) are computed will be application-specific

Let us fix some notation next. Expression sig_pk_(*m*) refers to the digital signature of message *m* with the private key *sk* that corresponds to the public key *pk*. The definition of SIG_pk_*i*__(*m*), given in figure 2, embeds the public key *pk*_*i*_ into the signed message. The notation 0.*H*(*m*) refers to the real number in the open interval (0, 1) obtained by interpreting *H*(*m*) as the mantissa of that real number over the binary representation of reals (base *b* = 2). As stated above, we make use of the concept of *Cryptographic Sortition*, an important technical ingredient of Algorand [6,7], to select a dynamically adjustable group of nodes for a specific task *task*, e.g. the next mining race. Formally, a node *pk*_*i*_ may perform task *task* at blockheight *r* if

2.2 $$0.H({\mathrm{SIG}}_{{\mathrm{pk}}_{i}}(r,\mathrm{task},Q^{r-1}))<\frac{n_{w}}{|PK^{r-k}|}$$

that is, if the hash of its signature of (*r*, task, *Q*^*r*−1^) is less than the quotient of parameter *n*_*w*_ and the size of the set *PK*^*r*−*k*^ of public keys that are—in principle—eligible to perform task *task*, based on the blocks from *B*^0^ up to *B*^*r*−*k*^, and possibly other access-control state as discussed below.

Note that this also reveals the interpretation of the security parameter *k*, a usage proposed in [6,7] already: public keys that only made their first appearance on the blockchain in the most recent *k* blocks must not and will not be selected for any tasks. This gives us a configurable way of controlling and mitigating Sybil attacks. In particular, this prevents an adversary from introducing such entities in more recent blocks in order to rapidly gain significant influence—which then may also increase the number of nodes it would control in the randomly selected mining nodes for blockheight *r*. We also point out that the current blockchain *B*^0^…*B*^*r*−1^ creates consensus for the values of the seed *Q*^*r*−1^ and expected size *n*_*w*_, and indirectly for the set of public keys *PK*^*r*−*k*^ used in (2.2).

Functions *getEligibleKeys*_pk_*i*__ and *getTransactionsForNextBlock*_pk_*i*__, shown in figure 2, refer to local state of node *pk*_*i*_: for the former, its local blockchain *B*^0^, …, *B*^*r*−1^, and for the latter a set of transactions (of the chain's application domain) that node *pk*_*i*_ has already seen and validated; the validation logic is also application-specific. Note that an implementation that uses machine learning to update system policy may make functions *getEligibleKeys*_pk_*i*__ and *getTransactionsForNextBlock*_pk_*i*__ dependent on the current state of such policy; for example, this would allow one to ban or add public keys, block or prefer certain types of transactions and so forth. Our White List is an example of such policy specification and enforcement. The pseudo-code below indicates which functions may want to extract values of local variables such as *k* and *n*_*w*_ from the blockchain.

Figure 2 shows additional functions, commented as (a)–(d) in the figure, we use as primitives:

(a) Specification of digital signatures for node *pk*_*i*_—where sig_pk_*i*__(*m*) refers to the digital signature of message *m* under the private key for *pk*_*i*_.
(b) Specification of checking whether node *pk*_*i*_ is eligible, in principle, to participate in task *task* (where task *mine* refers to a mining race) for the block with blockheight *r*: expression *PK* refers to a set of eligible public keys—typically a subset of the White List *L*.
(c) Function name for extraction of the set of eligible public keys from local blockchain state. The implementation of this function will also reflect the logic of the White List *L*.
(d) Function name for computing local transactions that should be in the next block (should node *pk*_*i*_ happen to win the mining race).

Computation of outputs for *getEligibleKeys*_pk_*i*__(*r*, *k*) in (c) and *getTransactionsForNextBlock*_pk_*i*__(*r*) in (d) will be application-specific.

**Figure 3.**
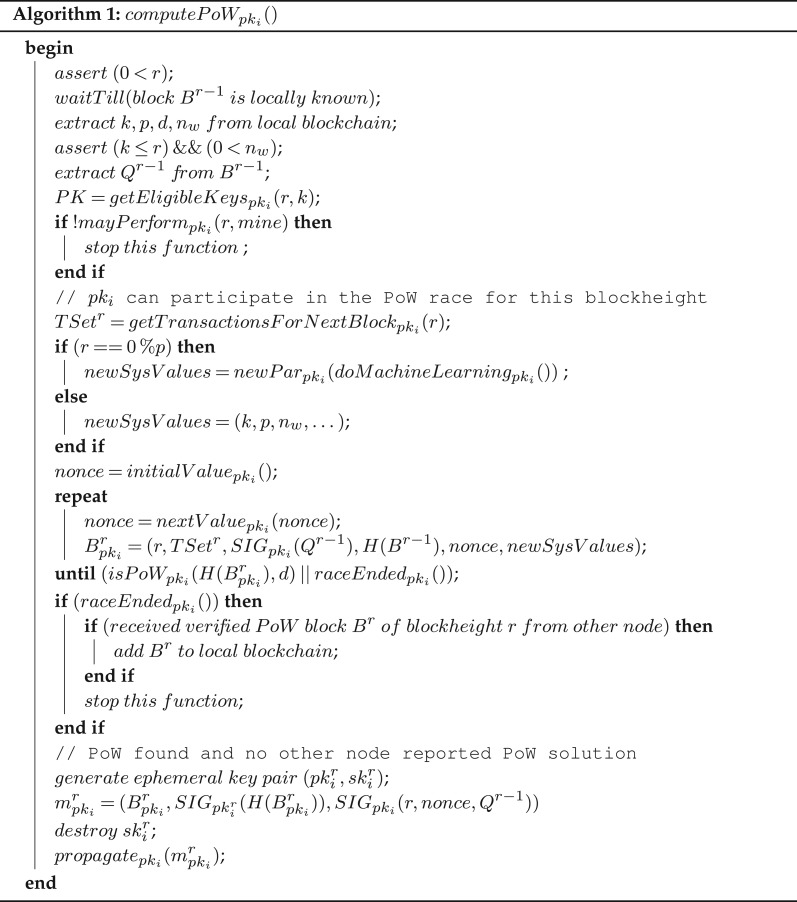
Specification of PoW for potential leaders for blockheight *r*. Nodes *pk*_*i*_ start mining only if they are eligible. New system values are the same as the old ones when *r* is not a multiple of *p*; otherwise, the new system values are determined by local machine learning.

In figure 3, we are now in a position to specify the code that nodes run to determine whether they are eligible to participate in the next mining race, and what tasks they will perform if indeed eligible. Node *pk*_*i*_ waits until it knows block *B*^*r*−1^. Then it extracts from the local blockchain the security parameter *k*, the period *p* at which machine learning and system parameter adaptation takes place, the level of difficulty *d* for PoW, and the expected size *n*_*w*_ of the set of nodes that are permitted to mine the next block *B*^*r*^.

Then it checks the integrity of these values, with appropriate exception handling (not shown). It also extracts the seed *Q*^*r*−1^ from block *B*^*r*−1^ and assigns to *PK* the set of all eligible public keys—a function of the local block state, security parameter *k* and the White List *L* (left implicit).

The function *mayPerform*_pk_*i*__ is then used with these computed inputs to determine whether node *pk*_*i*_ is indeed eligible to participate in specific task *mine*, i.e. the mining race for *B*^*r*^. Note that only node *pk*_*i*_ will know if it is eligible, assuming that it does not share the signature *SIG*_pk_*i*__(*r*, *Q*) with any other node: this signature is the input for the hash function *H*, whose output decides whether node *pk*_*i*_ is indeed eligible. In particular, no other node can produce this signature as it will not be in possession of the corresponding secret key *sk*_*i*_.

We note that an attacker who compromises node *pk*_*i*_ and so also knows the secret key *sk*_*i*_ will then know whether this node can participate in the specified task. However, this selection process is dependent on the seed *Q*^*r*−1^ in a non-predictable and non-manipulable way. Thus, the adversary would not know which nodes to compromise before the next mining race, machine-learning process, and so forth are about to start. This provides crucial resiliency to this blockchain system architecture.

Returning to the specification for mining, if node *pk*_*i*_ is not selected for mining the block with blockheight *r*, function *computePoW*_pk_*i*__ stops execution. Otherwise, node *pk*_*i*_ computes the set of transactions *TSet*^*r*^ to be included in block *B*^*r*^—dependent on the application logic for transactions. Next, it checks whether the block *B*^*r*^ is one in which system parameters may be updated according to machine-learning insights.

If so, it computes *newSysV* *alues*—a tuple of form (*k*′, *p*′, *n*_*w*_′, …)—as result of such machine learning (function *doMachineLearning*_pk_*i*__) and how these machine-learning outcomes inform decisions (function *newPar*_pk_*i*__) that modify system parameters. Otherwise, *r* is not such a periodic block, and then the value of *newSysV* *alues* defaults to those from the previous block *B*^*r*−1^ so that system parameters would not be adjusted in block *B*^*r*^.

Candidate blocks are computed as follows. The new seed *Q*^*r*^ is computed as the signature of the previous seed *Q*^*r*−1^. This ensures that the new seed is sufficiently random, and that an adversary cannot really influence the value of the next seed—in a model of an adversary that is similar to that used for Algorand in [6,7].

If PoW has been found (tested with function *isPoW*_pk_*i*__), the block (which contains the nonce for which PoW was found) together with a signature of its hash and a credential (a signature of the blockheight, nonce and previous seed) are sent across the network. Otherwise, node *pk*_*i*_ either exhausted its possible nonce values without finding PoW (and so stops the entire function); or node *pk*_*i*_ has learned from another node a verified PoW block *B*^*r*^ that is then added to his local blockchain, and its own mining efforts stop. Function *raceEnded*_pk_*i*__ captures the ability to detect either of these events (nonce space exhausted or verified block learned from another node).

Note that the message to be broadcasted contains a signature of the hash of this next block, but signed with an *ephemeral private key*—which also gets destroyed once the message has been sent. This ensures that an attacker who compromises nodes can no longer manipulate signatures of such hashes, for example, in order to recreate a segment of a chain assuming that it compromised the long-term public key *pk*_*i*_. The use of ephemeral keys requires a sufficient number of such key pairs such that other nodes can easily learn the corresponding set of public keys. There are standard solutions to this, for example, the use of identity-based public-key cryptography.

### 2.3. Optimization for initial system configuration

Let us now describe how the robust optimization framework in [8] can be used to compute initial system parameters. In that work, we assume that each miner has approximately the same hash rate in an enterprise blockchain system. We then formulate constraints, lower or upper bounds, on characteristics of the mining process. For example, we may stipulate that the average time it takes to find PoW for *s* miners at level of difficulty *d* and with nonces of *r* bits is within a given time interval. And we may want to ensure that the worst-case completion time for such a mining race is below a given bound with sufficiently small probability. We can then optimize over an objective that takes into account the procurement of mining machines, the energy costs of mining and so forth.

Optimal solutions then also tell us an ideal number of miners *s*_0_ and level of difficulty *d*_0_. We can then initialize *d* for the blockchain as *d*_0_ and set *n*_*w*_ = *s*_0_ as the expected number of nodes eligible to mine the next block. These values may become sub-optimal as the network evolves; for example, a consortium may deploy miners with heterogeneous hash rates over the life cycle of the blockchain. This is where the periodic machine learning is meant to adapt such system parameters.

## 3. Access control

We now discuss our approach to access control in blockchains based on PoKW, by focusing on the actions taken that change pertinent state of the blockchain system. These actions include:

(i) adding a block to the blockchain,
(ii) changing the value of one of the system parameters,
(iii) making possible changes to the state of smart contracts, and
(iv) initial system set-up of access-control state.

The first three items contain dependencies. In fact, adding a block may be seen as the basic mechanism by which actions, once granted, are enforced (in access-control terminology). For example, the action to change the level of difficulty from 12 to 10 would be recorded by writing the new value 10 into the new block. A policy may contain similar such rules, for example, that the value of *p* cannot be changed by much, to prevent an attacker from making such checkpoints occur too infrequently. Therefore, the validation logic that nodes use needs to reflect the access-control model: a new block that, within it, violates some aspect of the access-control model will be refused by honest nodes. For example, a policy *P* may say that changes of the level of difficulty cannot be greater than 10% of its current value—in which case the decrease of the value of *d* from 12 to 10 would not be validated.

The system parameters that actions may change include the expected size of committees *n*_*w*_, the level of difficulty *d*, the security parameter *k* for banning public keys freshly introduced in last *k* blocks from committees, and the value *p* that specifies that machine learning happens at each *p*th block creation. The latter system parameter is of particular interest to an attacker, since it controls the frequency with which such parameters, including *p* itself, may change. The access control for changing *p*, therefore, deserves special attention and needs to be very restrictive.

Actions that change the states of smart contracts, for example, the state of a White List *L*, would have to be consistent with the contracts' code, and so the validation of actions is here done by the consensus of running the code on the Ethereum Virtual Machine. Initial system set-ups may be dealt with in a completely different manner, discussed further below.

The access-control state of a PoKW-based blockchain, therefore, includes the following:

— the current blockchain of *r* blocks,
— the derived set of public keys *PK*^*r*−*k*^ whenever *r* > *k*,
— a policy *P*, in simplest form a set of rules, that is encoded in a smart contract, and
— a White List *L*, encoded in a smart contract.

Let us next discuss how the blockchain and its access control may be initialized.

### 3.1. Initial set-up

There seem to be two principal ways of initializing the access control for a PoKW-based blockchain: either appropriately adjust the body of function *getEligibleKeys*_pk_*i*__(*r*, *k*) in the cases in which *k* > *r* − 1 or let those who create the genesis block also create the initial part of the blockchain so that subsequent calls to *getEligibleKeys*_pk_*i*__(*r*, *k*) satisfy its assertion.

We now describe the latter approach in more detail. We have an initial value *k* > 0 that is set by those who are responsible for instantiating the blockchain and its initial configuration. In particular, those parties will create the first *k* + 1 blocks

3.1 $$B^{0},B^{1},\ldots ,B^{k}.$$

The creation of these first *k* + 1 blocks may use a much simpler access-control model during that phase. For example:

— The Genesis block contains initial values of system parameters, as well as references to smart contracts that encode initial values of an access-control policy *P* and White List *L*.
— The first *k* mining races are done by all nodes on that White List *L*.
— During these first *k* mining races, neither machine learning nor modification of system values or smart contracts that capture the state of *P* and *L* takes place.

The information in the Genesis block would be trusted by all parties. Consensus about that trust can be realized through means *external* to the blockchain itself. Such external mechanisms would also bootstrap trust into the first *k* + 1 blocks of the chain. Although this phase does not reduce mining to a kernel, it will neither consume much energy nor invite pooling behaviour in mining. This is so since the size of the initial White List *L* or of the initial value of *k* may be rather small.

For example, in a consortium of four parties, each one may supply a node to be on *L* and then *k* mining races take place between these nodes to seed the ‘chain security horizon’. In particular, the first *k* + 1 blocks may not even contain any payload information. Also, once the initial chain in (3.1) has been produced, the parties may form an external consensus that this chain is trustworthy as a ‘seed chain’ for the running PoKW system. Then, PoKW can operate as specified in §2.

Let us next consider how different types of tasks/actions shape access-control policy.

### 3.2. Action types and their access control

When mining blocks *B*^*r*^ with *r* > *k*, the system then switches to another access-control model, which is assumed to operate in a more hostile environment and so is more restrictive. And this more protected environment is realized through the adaptive PoKW blockchain itself.

It is important to note that information that impacts whether or not to grant an access is recorded or securely referenced on the blockchain: the White List *L*, the policy *P*, the set of public keys *PK*^*r*−*k*^ that appeared in the payloads of blocks

3.2 $$B^{0},B^{1},\ldots ,B^{r-k},$$

as well as the code that captures the validation logic of new blocks. In particular, any public keys that freshly appeared on the chain in the *k*-block segment

3.3 $$B^{r-k+1},B^{r-k+2},\ldots ,B^{r}$$

are not in *PK*^*r*−*k*^. We distinguish two types of access-control actions:

— Actions that change the state of one or several of the system parameters, e.g. a decrease of the level of difficulty or an increase in the expected size of the committee.
— Actions that change the state of the White List *L*.

We do not consider, at the moment, actions that might change the state of policy *P*. Rather, we view policy *P* as static since it enforces rules that ensure robustness of system stability. For example, policy *P* may be a ‘conjunction’ of rules, including the rules

— Addition of a block and its implicit change of access-control state shall never increase or decrease the size of *L* by more than 2% compared to its size at the last time its smart contract state changed.
— The level of difficulty shall not change by more than 10% compared to its previous value.^1^

The policy *P* is captured in a smart contract, and may even be controlled by a central entity—depending on the nature of the use case. Let us next discuss how the White List may be realized.

### 3.3. Different realizations of the White List *L*

We may think of the White List *L* as a qualitative list, telling us those nodes that are definitely on that list. Alternatively, we may have a threshold *th*_*L*_—which would itself be a system parameter encoded in a smart contract or in the block structure. Then, White List *L* would be a mapping from nodes to reals. The logic would then be that *L* represents the set of network nodes *n* that satisfy

3.4 $$L(n)>th_{L}.$$

In the former case, access-control actions may simply be to add or remove nodes from *L*. In the latter case, such actions may increase or decrease *L*(*n*) based on evidence from machine learning, say. For example, if the periodic anomaly detection flagged up node *n*, an action may decrease *L*(*n*) as a function of the computed anomaly score.

A question in that context is how such an action would be validated by other nodes. It could simply be approved, if the node was eligible to do the learning task. But one can imagine more complex validation logic, e.g. that the proposed decrease is an agreed upon deterministic function of the anomaly score, and there may perhaps even be some validation of that score itself. Related to that, equation (3.4) may be instantiated with thresholds that are specific to tasks, for example, separate thresholds for mining, anomaly detection and network stabilization. However, more complex realizations of the White List *L* potentially offer an attack surface and would have to be formally modelled and verified.

### 3.4. Policy-based access control

It is customary to express policy-based access control within an architecture. For example, in XACML [10] a policy decision point (PDP) is the system part that computes whether a particular action (i.e. an access request by some actor) should be granted. The policy evaluation point (PEP) would then operationalize that decision on the system, for example, to perform the action if granted or to log a denial if the action is not granted. We may think of the PDPs and PEPs as decentralized engines, as seen, for example, in the so-called User Managed Access Control as developed by the Kantara initiative.

We now want to understand how this view of access-control systems can be interpreted in the PoKW context. For the first *k* + 1 blocks of the system, the PoKW system would simply implement whatever access-control logic this initial phase of system set-up might have. After *k* + 1 blocks have been created, the PDP might operate a follows. Let node *n* make a request to perform action *a*. The PDP then reasons as follows:

R1 If *n* is not in the set *PK*^*r*−*k*^, then the action is not allowed.
R2 Otherwise, if *L*(*n*) ≤ *th*_*L*_, then the action is not allowed.
R3 Otherwise, if action *a* violates any of the rules in policy *P* in its current state, action *a* is not allowed.
R4 Otherwise, if node *n* is not in the sortition committee for the task associated to action *a* at the local blockchain state, then action *a* is not allowed.
R5 Otherwise (subject to refinement in particular circumstances and for certain actions), action *a* is allowed.

In the priority composition of the five rules R1–R5, the three components *PK*, *P* and *L* of access control as well as the cryptographic sortition are semantically a conjunction for granting access: all of them need to rule that the action *a* is allowed in order for action *a* to be permitted.

As for the PEP, in a blockchain context this mostly refers to the validation logic for new blocks, which each node would independently verify. For example, the action of node *n* of proposing a new block has task type *mine* and so this would only be accepted if *n* could be in the mining committee of that race according to the above five rules, for action *mine*.

However, we need to be careful that this model does not introduce feature interactions that lead to inconsistent access-control state, and so to a deadlock in the system. For example, the function *getEligibleKeys*_pk_*i*__ should in its body compute a set of public keys that only contain nodes *n* satisfying *L*(*n*) > *th*_*L*_. Otherwise, use of cryptographic sortition with *n*_*w*_ = 4, say, may compute with non-trivial probability a set of four nodes—none of which satisfy *L*(*n*) > *th*_*L*_. In that case, the validation logic for new blocks, which subsumes the checks of the access-control logic, would simply refuse to validate *any* new block.

We turn to describing how committee memberships are determined for the concrete specification in §2.

### 3.5. Determining node eligibility

Let us illustrate how the set of eligible public keys may be computed after *k* + 1 blocks have already been created and when there are only two possible tasks:

— *mine*: the new block *B*^*r*+1^ is such that *r* + 1 is not a multiple of *p*, and so no learning or other system parameter adjustments will take place in that creation
— *mine*_*and*_*adapt*: the new block *B*^*r*+1^ is such that *r* + 1 is a multiple of *p*, and so the new block not only needs to be mined, but also system parameters need to be adjusted based on anomaly detection.

To make things simple, we also assume that the policy *P* has no influence over which nodes are eligible for this new block creation; *P* will only constrain the possible sets of parameters. Let us also assume, for sake of further simplicity, that all system parameters stay constant for *p* − 1 consecutive blocks once a *mine*_*and*_*adapt* task just created a new block. The code for function *computeEligibleKeys*_pk_*n*__(*r*, *k*) would then produce the following output:

3.5 $$computeEligibleKeys_{{\mathrm{pk}}_{n}}(r,k)=PK^{r-k}\cap \{n^{\prime }|L(n^{\prime })>th_{L}\}.$$

Node *n* can then call function *mayPerform*_pk_*n*__, with either task *mine* or task *mine*_*and*_*adapt*, to determine whether it is eligible to participate in the mining race for *B*^*r*+1^. Note that node *n* has no incentive to call that function with the wrong task: block validation will invariably check whether the node that mined this block was eligible for the unique task of that new block.

Finally, we make some points about how our approach affects the validation of new blocks.

### 3.6. Validation logic of new blocks

For task *mine*, the structure of the new block would be such as expected by the logic of the payload transactions and that of the creation of the new seed, whereas other system parameters would not be allowed to change. This implicitly determines the validation logic for new blocks of type *mine*. For new blocks of type *mine*_*and*_*adapt*, the validation logic inherits the logic for the transaction sets and new seed, but would also verify that the system parameter values meet the policy *P*.

The policy *P* needs to have a deterministic semantics so that a vector of system parameter values, as the suggested new values in the block *B*^*r*+1^, is either accepted by the policy *P* or rejected. Rejection would mean that the entire new block *B*^*r*+1^ does not validate. This also implicitly specifies how other nodes validate such a new block.

## 4. Public Proof of Kernel Work networks and democracy

It is well understood that PoW, as used in Bitcoin, led to the centralization of mining into pools. The interplay of game theory, adjustment of level of difficulty of PoW, and exchange value of mined Bitcoins also resulted in a dramatic increase in the level of difficulty. Bitcoin, therefore, consumes way too much energy, on par with an entire economy such as Sweden. It also means that ordinary nodes and devices are essentially deprived from the ability to ever mine successfully. Moreover, the mechanisms for sharing rewards within pools are complicated and cannot prevent nodes to participate in more than one pool and to gain an unfair advantage. The latter problem is similar to the known Dilemma of the Commons; see, for example, the discussion in [11].

So what can PoKW offer in this problem space? It seems that it can at least provide a reduction in the energy consumption of PoW in the network: equation (2.2) guarantees that an expected number of *n*_*w*_ nodes will be able to mine the next block. However, this assumes that public keys are somehow bound to individual mining units. An attacker may use mining rags to duplicate and scale up such mining for eligible public keys. This means that PoKW-based blockchains that contain considerable monetary incentives for mining are likely to see more energy consumption caused by such mining behaviour.

In applications in which identity management is an integral aspect of the blockchain, public keys may be generated from identities and one could manage, in function *getEligibleKeys*, how many public keys of a given identity would be classified as eligible by that function. In fact, consider *P*_*a*_ as the set of private keys that agent *a* knows and that function *getEligibleKeys* decides to be eligible. Then, the size of set *P*_*a*_ may be seen as a relative stake of agent *a* in determining the next block. And this stake is independent of the hash rates that agents may employ once they are permitted to participate.

We note that the unfairness that may result from the different hash rates of, say, a generic smartphone of a typical user of a public network and of a powerful miner, may be less pronounced for lower levels of difficulty. This is so since the propagation times for blocks on the network may influence the leader election in a peer-to-peer gossip protocol, for example.

One concern with PoKW, however, is that if even one adversarial miner with powerful hash rate is eligible to mine the next block, then the adversary can take over control of the block mining. Let us understand this issue better though. Recall that the probability of finding PoW within time *t*, at hash rate *r* (the number of PoW attempts done per second), and for level of difficulty *d* is exponentially distributed:

4.1 $$Pr(Success_{r}\leq t)=1-e^{-rt/d},$$

where *Success*_*r*_ is the actual time for a miner with hash rate *r* to find a PoW. Let us illustrate how one may analyse an attack that uses machines with high hash rates if the benign part of the network has only half as many machines with such higher hash rate available.

We consider *r* = 10^9^, so the high hash rate is 1 GHash per second for both attacker and benign node. Let us say we have 1000 nodes in the network of which 200 are controlled by the attacker *and* have hash rate *r*. Let us assume that 100 nodes in the network also have hash rate *r* but are *not* controlled by the attacker. For the expected size of the mining committee, we choose *n*_*w*_ = 50, and suppose that *p* = *n*_*w*_/|*PK*^*r*−*k*^| equals 0.1.

This means that *n*_*a*_ = 50 × 200/1000 = 10 nodes of those that are controlled by the attacker and have hash rate *r* are expected to be eligible to mine. For *d* = 16, say, this gives us the probability

4.2 $$Pr(AttackSuccess_{r}\leq t)=1-e^{-(t/d)rn_{a}}=1-e^{-t\times 10^{9}\times 10/16}$$

that the attacking *n*_*a*_ nodes find PoW within *t* seconds. Similarly, we have *n*_*b*_ = 50 × 100/1000 = 5 as the expected number of benign nodes that are eligible to mine the next block and have hash rate *r*. From this, we may compute

4.3 $$Pr(BenignSuccess_{r}\leq t)=1-e^{-(t/d)rn_{o}}=1-e^{-t\times 10^{9}\times 5/16}$$

as the probability that these benign nodes find PoW within time *t*. We can see from this that both of these probabilities are exponentially distributed. Moreover, their rates reflect how many more compromised machines an attacker has among those machines with the high hash rate: the attacker has twice as many such machines, and so the rate in (4.2) is twice the rate in (4.3).

Let us illustrate how this translates into concrete probabilities. Table 1 shows these probabilities for a few values of *t*. It is worth noting that, for smaller time periods, these probabilities reflect well how many more powerful machines the attacker controls. However, for longer time periods, these differences become much less pronounced. As already discussed, the time differences in this table may not matter too much within a PoKW-based network, since *PoKW* solutions need non-negligible time to propagate on the network.

**Table 1.** Some values of the probabilities in (4.2) and (4.3), rounded to three significant digits. Probabilities refer to a group of nodes finding PoW, not winning a PoW race that includes convincing the network that one has won that race.

*t*	*Pr*(*AttackSuccess*_*r*_ ≤ *t*)	*Pr*(*BenignSuccess*_*r*_ ≤ *t*)
10^−8^	0.998	0.956
10^−9^	0.465	0.268
10^−10^	0.06	0.03

We think that PoKW may also help with making public networks, in which the mining does not generate significant monetary value to the miner or her pool, more democratic. For example, one may generate a small donation for a good cause and the controllers of such a network may use smart contracts to manage the list of organizations to which one may donate.

Furthermore, the creation and state of such a list may be determined through participation of the network's social agents, where common social values would influence which organizations to propose, elect and perhaps remove. Such a socio-computational model is particularly attractive for lower levels of difficulty, and where these levels may neither change much nor exceed a threshold that would discourage participation of small devices.

A combination of PoKW with other consensus mechanisms may be used to control the ability or incentive of nodes to duplicate eligible key pairs on other devices; see, for example, the discussion in [3] on how one may combine PoKW with Proof of Elasped Time.

## 5. Attack vectors and their mitigation

We now discuss some potential vectors through which an adversary or a group of adversaries could attack this system, and how we may mitigate such threats. First, let us state the underlying security model.

*Security model*: The overall security model is that all nodes trust the current blockchain that they know, and may not trust anything else. Also, we assume that an adversary or group of adversaries cannot compromise more than a certain percentage *pc* of *all* network nodes, at any given point in time.

Let us analyse the use of Cryptographic Sortition. We assume that the initial seed *Q*^0^ in the system's Genesis Block *B*^0^ will be generated by a cryptographically strong pseudo-random generator. If the Genesis Block is created by a central system authority, the latter could create the seed for the generation of *Q*^0^ from a high-entropy source. One could alternatively generate this seed using decentralized protocols for verifiably generating secrets, e.g. the JF-DKG protocol [12], if there is a well-defined initial set of nodes that would participate in this process. The latter approach may produce more trust into the initial block data *B*^0^, including the value of *Q*^0^. Similar considerations apply for the creation of the next *k* blocks on the chain.

The generation of subsequent seeds *Q*^*r*^ is such that it is a deterministic function of the previous seed *Q*^*r*−1^—whose value we trust based on the consensus of the current blockchain—and of the digital signature of some node *pk*_*i*_. An adversary cannot change the format of this new seed since block validation would otherwise fail. An attacker may only try to create this signature with a key pair (*pk*_*i*_, *sk*_*i*_) of his choice, assuming he is able to compromise a certain percentage of all nodes.

But all such signatures would use a specific algorithm, in our use case this is ECDSA for a particular Elliptic curve. And we assume that an adversary cannot exploit any changes in digital signatures based on changes of private keys. This is a reasonable assumption as the creation of signatures acts like a (key-dependent) pseudo-random generator.

Let us now consider how an adversary could exploit knowledge of the new seed value *Q*^*r*^, for example, in a setting in which the adversary would first learn the new block *B*^*r*^ and could ensure that other nodes receive this block only after some delay. For those nodes which the adversary has already compromised, we may assume that the adversary knows the private keys of these nodes. Assuming that the set of public keys *PK* that are, in principle, eligible to participate in a mining race for *B*^*r*+1^ is computable from the current blockchain, the adversary therefore can evaluate the entire body of *mayPerform*_pk_*i*__(*r*, *mine*) for compromised nodes *pk*_*i*_ to determine which of these nodes are eligible to mine.

However, if he has not yet compromised a node *pk*_*j*_, then he would not know its secret key *sk*_*j*_ and so he cannot evaluate the body of *mayPerform*_pk_*j*__. Therefore, the attacker cannot know which of the nodes that he has not yet compromised will be able to participate in the next mining race. This means that he has no additional information that may help him determine which additional nodes to compromise next—assuming that such actions take effort and time and can only be done for a limited number of nodes. Therefore, an adversary may as well resort to compromising nodes randomly for the purpose of influencing mining races.

This means that he has to compromise a percentage *pc* of *all* network nodes in order to guarantee that he compromises an expected percentage of *pc* of eligible nodes for each mining race. To make this concrete, assume that *n*_*w*_ equals 50 and that there are 100 000 nodes in the network. Then an adversary needs to compromise 10 000 nodes if he expects to compromise five of the 50 nodes that will be eligible to mine the next block.

Another threat is that an adversary who knows a private key *sk*_*i*_ can duplicate this key across fast PoW devices to engage in a private, parallel thread of the current mining race. Depending on the number and types of devices used for this, the adversary may gain a considerable advantage in winning the mining race. Specifically, the probability that the adversary will compromise at least one node that is eligible to mine the next block can be seen to be equal to

5.1 $$1-{\left(1-pc\right)}^{n_{w}}.$$

For example, for *n*_*w*_ = 50 and *pc* = 0.1 this gives us a probability of 0.99484622479268 that the adversary controls at least one of these eligible nodes. However, the discussion around table 1 suggests that this may not be a grave concern whenever the public network has sufficiently many benign nodes of similar hash rates available. This is certainly less of a concern in an enterprise setting.

Another threat stems from the ability to propose empty, sparse or low quality transaction sets *TSet*^*r*^ within blocks *B*^*r*^. We assume that the system policy, embedded into the blockchain, would be able to flag up such behaviour so that the machine-learning engines could react to this—for example, by removing a node from the White List *L* or by adjusting system parameters as a corrective response.

Naturally, these control mechanisms themselves, including the machine-learning algorithms used, may be subject to attack and manipulation. Adversarial machine-learning techniques (e.g. [13]) may be mitigated against, given that most if not all the data for learning would stem from the current blockchain, which we would trust in our security model, and so data poisoning would be prevented. Other control mechanisms, including those based on policy, would need to be internally consistent so that they would not offer denial-of-service type attacks on their own access-control logic. Formal verification has already been applied extensively in the area of access-control policies, and such validation could therefore also be done for smart contracts that embody such policies.

The strength of smart contracts, as compute engines that are immutable and for which there is system consensus, also reflects their weakness: the limited ability to manage change. This needs to be reconciled with support of the entire life cycle of a system. One may achieve this, for example, through voting mechanisms within smart contracts, similarly to how Bitcoin handles major system configuration changes through a vote of miners. We think that such voting mechanisms will offer an ability to recover from major system incidents, should an attack of an adversary ever produce systemic damage that requires stability-preserving system repair.

## 6. Use case

We report here a pilot project in IoT and mobility, where PoKW and our approach above were used. The outcomes of this pilot are illustrated in this short video:

https://www.youtube.com/watch?v=KvyF78RTj18&feature=youtu.be

The pilot was run by XAIN—a University of Oxford spin-off focused on strengthening the blockchain through reinforcement learning and PoKW as discussed above, to enable flexible, efficient and secure machine communication networks. The use of PoKW allows for massive energy reductions and network democratization that directly includes mobile low-power devices, integrated in embedded systems, e.g. in engine control units (ECUs) in machines or connected cars.

The benefits of this work and innovation are rooted in realizing the following abilities, of interest to owners of a Porsche car:

— record traffic data over the blockchain, in direct communication with other vehicles,
— lock/unlock your car, way faster and much more secure, thanks to a blockchain-powered direct offline connection (no server connection involved),
— grant somebody else temporary access to your Porsche, even if you are nowhere near,
— receive real-time notifications about who/when/where accesses your Porsche,
— get mail packages securely delivered directly inside the trunk of your parked car, and
— obtain resilient security of a fully decentralized blockchain implementation.

Additionally, Porsche itself gains direct benefits on the basis of data access, including:

— an increased trust in vehicle data, entirely audited and usable for reports, certificates and especially the access of local data for predictive maintenance and autonomous driving,
— an increased security for vehicle software, used for flexible access transactions and secure over-the-air update opportunities,
— the XAIN blockchain as a distributed, lightweight and trusted ‘app store’ for fast integrations of third-party software, such as from DHL,
— an increased customer trust, while being GDPR compliant for data usage, and
— the potential training of distributed machine learning to achieve better local models for autonomous driving, thus supporting a knowledge transfer platform.

The latter is a key point, privacy-sensitive computations are done locally (within a company or even within a vehicle), and only statistical results are shared to improve overall learning and analytical insights.

This use case needs to emphasize safety and security as key consideration. Unlocking your Porsche car happens via an online transaction that is mined by the vehicle network, a public permissioned blockchain with hybrid nodes inside the car. Each node mines with PoKW, developed as an energy-optimized consensus algorithm that works in ECUs of the cars. The running network is then stabilized through reinforcement learning.

### 6.1. System architecture

**Figure 4.**
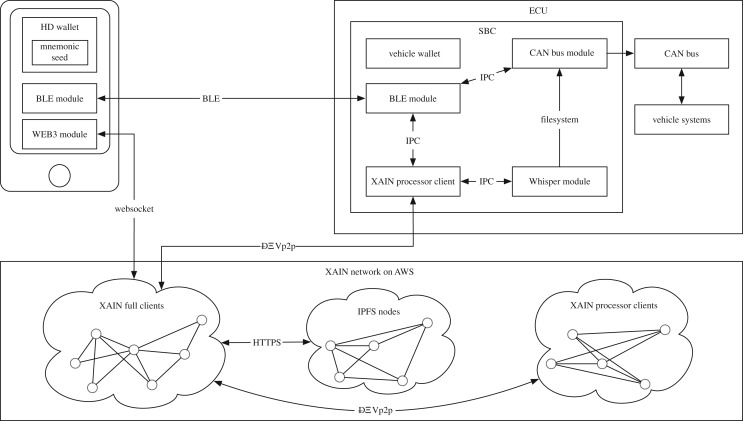
System architecture of our pilot use case for Porsche: user smartphone components are shown on upper left; vehicle ECU, CAN bus and vehicle systems are depicted on upper right; and the XAIN network is seen below. Edges are labelled with communications and their realizing technology.

The pilot system comprises a variety of different network and system components that refer to the architecture diagram shown in figure 4. Inside the car, we run a single-board computer (SBC), which includes a XAIN Processor with the ability to process data (mining) and to store the most important information. Further, the SBC stores the car wallet with the encrypted private keys for signing transactions and for encrypting all communication via Bluetooth Low Energy (BLE), a BLE module as an implementation of the transaction protocol for communication with the smartphone, a Whisper module as a message protocol for secure transmission of encrypted vehicle data, an LTE module for communication between the processor with the blockchain network, and a CAN bus module as the interface between the SBC and the vehicle itself.

Subsequently, we implemented a smartphone app, which includes a digital wallet (HD Wallet) as an Initiator Client that comprises the encrypted private keys, necessary for signing transactions, for the encryption of the communication via BLE and for the encryption of the symmetric key of the vehicle data. This allows for all keys to be recovered via a mnemonic seed.

Thus, if one loses the seed, all data are lost, a security and privacy requirement—especially when considering GDPR. Moreover, the app comprises a BLE module for communicating with the car, plus a Web3 module for the communication between the app and the different XAIN clients (processors or dataloggers) and for requesting smart contract data or for sending signed transactions.

Last but not least, we implemented a network of multiple nodes on AWS with three different categories. Firstly, Inter Planetary File System (IPFS) nodes, which store the encrypted vehicle data, transferred via Whisper. Secondly, dataloggers that each contain the complete Merkle tree of the blockchain as a security instance. These nodes are also used as so-called boot nodes for the hybrid nodes in the car. Thirdly, we also implemented a network of processors (miners) on AWS that perform the mining process (PoKW) and store less information over time (recursive pyramid data structure). The processors can be removed at a later stage from AWS, once enough cars have running processors installed and the network becomes large enough.

### 6.2. Smart contract architecture

The architecture of the pilot system is based on three main smart contracts (among others): UserRegister, UserHistory, and VehicleState.

— UserRegister is just what the name says; it provides an overview of all users of the system. It allows queries as to whether an address represents a user and the possibility to exclude users from the system (ban from the White List). It also allows querying the address of a user's UserHistory contract.
— UserHistory contains the hash values of the driver's history (driving history). The actual records are encrypted on the distributed file system IPFS. The contract contains the addresses of the vehicles that are allowed to add entries in the driver's history of the user. These vehicles are the ‘authorized cars’ for this user. It is also possible to deposit access permissions that allow outside parties access to specific records.
— VehicleState represents the state of a vehicle. Mainly, this means an assignment to an owner, as well as a mapping of addresses to permissions. These permissions determine which specific users have rights to the vehicle. For example, when opening the doors, the trunk or starting the engine. These authorizations are not fixed but are represented by an identifier that is interpreted by the above-described application scenarios. Authorizations are always provided with an expiration date but can be manually withdrawn at any time.

### 6.3. Access control and key management

**Figure 5.**
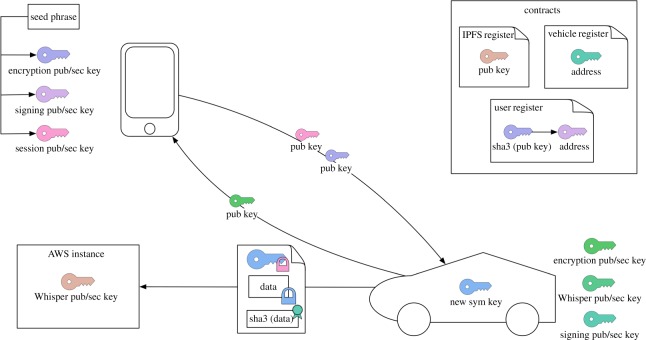
Schematic overview of our approach to key management in our pilot use case.

The diagram in figure 5 shows a different view of the system architecture, focusing on the management of access control and cryptographic keys. Overall, the XAIN network and this pilot system are using established, tried and tested, cryptographic primitives—and in standard usage modes. We stay well clear from ‘rolling our own cryptography’ in order to make our systems as secure as is possible by the state of the art in cryptography.

Essentially, access is granted when an appropriate and verifiable cryptographic credential is being presented that matches the request to the sought access, e.g. a specific action performed by the vehicle.

We follow best security practice by using different private/public key pairs for different intents of the same user (a smartphone, the SBC or other system agents). In particular, keys for symmetric encryption of payload data, such as the data generated by driving behaviour, are encrypted with a private key dedicated for communicating such symmetric keys. Whereas the signature of transactions or messages is performed by another private key. Certificates are generated by digital signatures that involve the use of standard hash functions, in this case SHA-3.

Our security protocols for key and message exchange, sketched below, adapt common protocol primitives and steps to the particular tasks at hand. For example, we rely on the generation of nonces (which is security-sensitive code that needs to guarantee that nonces never get regenerated, in particular, that the state of that generator does not ‘flow over’ to an initial counting state).

Cryptographic credentials such as public keys are stored in smart contracts, a register that functions as a secure public-key infrastructure that is resilient to adversarial manipulation to the same extent that smart contracts are resilient to unauthorized modifications. The private keys for the wallet in a smartphone, that is the private keys that belong to a human agent who wishes to interact with the vehicle, are all determined by a mnemonic seed—giving us a good balance of security and usability, and also a sole and simple source whose destruction also ‘destroys’ (knowledge of) these private keys.

### 6.4. Direct connector via Bluetooth Low Energy

We now describe the pertinent steps of communication protocols. The owner can communicate using the smartphone app, encrypted via BLE with the vehicle. The following actions are currently available: opening, closing the vehicle and opening the trunk.

Both the user and the vehicle have two private key types. A key is used for encrypted communication via BLE, here called Type 1 key. The other key (Type 2) is again used only for signing transactions and messages.

The transfer protocol that turns requests into actions works as follows: the user connects to the vehicle securely via the smartphone app. The smartphone app and the vehicle exchange their Type 1 public keys. After the vehicle has received the Type 1 public key of the user, it checks whether the key is contained in the UserRegister smart contract. However, the Type 1 public key is not contained in the smart contract, rather only the SHA-3 hash of this key.

If the Type 1 public key exists, the smart contract returns the XAIN network address of the user—the shortened SHA-3 hash of Type 2 public key. Subsequently, a random nonce is generated and sent back encrypted to the smartphone app with the Type 1 private key of the vehicle and with the Type 1 public key of the user. The random nonce is used to prevent replay attacks.

In the smartphone app, the random nonce is decrypted with the Type 1 private key, incremented by 1, encrypted with the Type 1 public key, and sent back to the vehicle along with a time stamp and the selected actions (e.g. a request to open the vehicle). The action and the time stamp are signed with the Type 2 private key of the user. The vehicle first checks to see if the nonce has been successfully increased by 1.

Subsequently, it verifies whether the signature of the requested action matches that of the user's XAIN network address. Finally, it is verified whether the time stamp is within the specified time interval and whether the user has the authorization to perform this action. The verification of the authorization is technically realized by a call to the VehicleState smart contract. The outcome of that verification activity is then recorded via a transaction (signed with the Type 2 private key of the vehicle) in the smart contract.

The so-called ‘Eventual Consistency’ property of the blockchain makes these actions available even when the vehicle is not connected to the Internet. However, there are restrictions on the validity of third-party permissions in such circumstances, as discussed in the next subsection.

### 6.5. Remote control of vehicle access

Via the smartphone app, certain actions (opening and closing the vehicle, opening the trunk, assigning or withdrawing authorizations) can also be carried out remotely. For that, the smartphone app communicates via the Web3 module with the distributed clients. Once the user selects an action through the app, a transaction with the selected action is created and signed with the user's private key on the smartphone. After signing, the transaction is sent to the network nodes.

The ‘open’, ‘close the vehicle’, ‘open the trunk’ actions are all time-stamped to ensure that past actions or actions planned for the future are not performed at present. The authorizations, however, may refer to the future, but their validity is limited in time. The validity depends on the difference between the last synchronization of the blockchain client and the network. If the difference is too large, permissions to third parties are also ignored for security reasons. The owner could have already withdrawn long ago a permission during this time interval. Only the owner can open the vehicle at any time with the smartphone via BLE (without Internet connection). The vehicle smart contract can be easily extended with further actions.

### 6.6. Secure data logging and auditing

If the owner opens the vehicle, the public key responsible for encrypting the vehicle data is sent to the SBC via BLE. The SBC is connected to the vehicle via the CAN bus interface. Once the engine of the vehicle starts, the SBC logs the generated vehicle data. The logged vehicle data are stored in small files (chunks), encrypted on the data system of the SBC.

The data are encrypted with a symmetric key, randomly generated by the vehicle. The symmetric key is then encrypted with the owner's public key and attached to the file in the form of metadata. The data package (consisting of the encrypted vehicle data, the encrypted symmetric key and further metadata, such as the time stamp of the file) is then sent via the Whisper protocol V5 to the XAIN clients. As an identification of the data package, a SHA-3 hash is formed via this data package and signed with the private key of the vehicle. The data package itself is encrypted with the public Whisper key of the AWS client. This public key is stored in a smart contract (a register for AWS clients connected to an IPFS node).

Only these AWS clients can decrypt this data package. Public keys can only be added or removed by Porsche itself. As soon as an AWS client receives a data package, the sender's signature is checked with the VehicleRegister.

If a vehicle with this address exists, the data package is stored in IPFS and the resulting multi-hash, signed with the private Whisper key of the AWS client, gets sent back to the sender. The multi-hash is subsequently stored by the SBC via a transaction in the UserHistory contract of the owner. The AWS clients are stateless. If the SBC does not receive a response after a specified time interval, the data package is resent. The time intervals can be freely selected.

In our live test drive, we chose a transmission interval of 2.5 s and were thus able to follow the driven route via our smartphones almost in real time. For the streaming of data, we used the functions and possibilities of IPFS. It is interesting to note here that Swarm could be an alternative to IPFS/Whisper. But we did not use it, because Swarm is still in a very early stage of development.

However, all XAIN system components are loosely coupled, which means that we can easily replace the IPFS/Whisper system with Swarm at a later stage. The entire data logging case provides the network with the benefit of a very secure, flexible, immutable and, most importantly, GDPR compliant means to collect valuable data. Data can be used by Porsche for third-party integrations on the platform, as well as for a personal revenue stream on the basis of reports. One such example is a trusted car certificate that informs about the history of a vehicle, including not only information such as mileage but also past driving behaviour, which can be used as a trusted certificate when re-selling the car. Furthermore, the data on their own are also highly valuable to Porsche itself when it comes to predictive maintenance and autonomous driving, both involving not just global models but also the locality of data.

### 6.7. Third-party integration

One of the XAIN pilot system's main features is its flexible utilization as a distributed app store for vehicle software by third parties. Third parties can access the Porsche system as soon as Porsche will have published its system and will then allow the integration of third-party addresses in the UserRegister smart contract. To communicate with the vehicle, a third party only needs two private keys and an implementation of the BLE transmission protocol. If the owner of the vehicle allows a third party, say DHL, access to the vehicle data, further interesting use cases are possible. For example, a DHL employee can not only open the trunk, but can then also determine the last location of the vehicle and thus deliver the package easily and quickly.

Further use cases include telematic insurances, fleet management, electrical charging and entertainment applications or over the air system updates in general. In the case of telematic insurances, for example, Allianz can ask the vehicle owner via the smartphone app, whether it may access the vehicle data for a smart contract as a telematic insurance that evaluates driving behaviour, such that the owner only pays per usage and pays less when exhibiting good driving behaviour. If the owner agrees, the encrypted symmetric key used to encrypt the vehicle data is decrypted with the owner's private key and then re-encrypted using the Allianz public key. Subsequently, this encrypted key will be sent to Allianz.

These suggested use cases are just an extension of the underlying product's wide range of capabilities. With the help of the blockchain, we have created a platform that makes it possible to safely communicate with a vehicle from the outside. The advantage of this platform is that it allows building applications without requiring their own security stack and/or additional hardware. This brings interaction with the connected car to the level of a smartphone, where the user has constant and immediate access to the newest standards with a quick software update.

### 6.8. Open access of source code

The pilot was done for a commercial partner and so we are unable to offer open access to its source code. However, we are in the process of building the XAIN protocol as a flexible and expressive framework for policy-based, and user-centric access control and where owners of resources may delegate not just access but also the ability to write policies. We envision that, in the future, the code base of this approach—including tool support for writing, verifying and compiling access-control policies—will be made available to the general public. This will, therefore, allow parties to program applications that create resilient services through fine-grained access control enforced by the XAIN protocol.

## 7. Conclusion

In this paper, we presented a refinement of PoW, PoKW, as a secure means of reducing the number of public keys that are eligible to participate in the next mining race. The paper also offered thoughts on how this approach may reduce the overall energy consumption of the blockchain network and how it may support attempts of making public PoKW-based networks more participatory and democratic. We further discussed possible attack surfaces for PoKW-based blockchains, and means for mitigating against such attacks. Finally, we reported on a use case in mobility and IoT that integrated PoKW into the Ethereum technology stack, as maintained by XAIN AG for its enterprise systems.

In future work, we mean to advance the work reported in this paper through cooperation with commercial partners that have an interest in building and maintaining more democratic and participatory public blockchains, especially in the Internet of Moving Things. There is a lot of commercial interest and willingness to engage in that space as evidenced, for example, by the Mobility Open Blockchain Initiative (www.dlt.mobi).

## Footnotes

1 Or perhaps as a function of those values from the last *p* blocks.

## Data accessibility

This article has no additional data.
